# Supplementary material for: Cellular network entropy as the energy potential in Waddington's differentiation landscape
Source: Sci Rep. 2013 Oct 24;3:3039. doi: 10.1038/srep03039 (PMC3807110; doi:10.1038/srep03039)
Supplement: Supplementary Information [file srep03039-s1.pdf]

Supplementary Information:

Cellular network entropy as the energy potential in Waddington's differentiation landscape

Christopher R. S. Banerji [1,2], Diego Miranda-Saavedra [3], Simone Severini [2,4], Martin Widschwendter [5], Tareq Enver [6], Joseph Zhou [7] and Andrew E. Teschendorff [1,2,8*]

[1] Statistical Cancer Genomics, Paul O'Gorman Building, UCL Cancer Institute, University College London, 72 Huntley Street, London WC1E6BT, United Kingdom, [2] Centre for Mathematics and Physics in the Life Sciences and Experimental Biology, University College London, London WC1E 6BT, United Kingdom, [3] Bioinformatics and Genomics Laboratory, World Premier International (WPI) Immunology Frontier Research Center (IFReC), Osaka University, Osaka, Japan. [4] Department of Computer Science, University College London, London WC1E 6BT, United Kingdom. [5] EGA Institute for Women's Health, University College London, London WC1E 6BT, United Kingdom, [6]UCL Cancer Institute, University College London, 72 Huntley Street, London WC1E6BT, United Kingdom, [7]Institute for Systems Biology, 401 Terry Avenue North, Seattle, WA 98109-5234, USA, [8] CAS-MPG Partner Institute for Computational Biology, 320 Yue Yang Road, Shanghai 200031, China.
[*] Corresponding author: a.teschendorff@ucl.ac.uk

Supplementary Materials & Methods + Supplementary Figures S1-S29.

Expression database

We compiled an expression database, consisting of over 800 samples, from the sources below. The choice of data sets was guided by our desire to perform specific comparisons between certain cell types (e.g. comparing hESCs to MSCs, or comparing CSCs to normal stem cells), thus requiring, if possible, that these specific cell types were profiled as part of the same study, in order to minimise potential confounding by batch effects. In the case of Affymetrix data, if RMA normalised data was provided on the authors website, or as part of the GEO submission, this was used. If normalised data was not provided, or if this was unspecified, raw data was downloaded and RMA normalised using the Bioconductor affy package. In the case of Illumina data sets, the normalised data provided by the respective studies was used. In all cases, quantile normalisation was performed to normalise across all arrays within a study. Finally, for later integration with the protein interaction network, expression profiles of probes mapping to the same entrez gene IDs were averaged. Probes mapping to multiple genes were excluded.

The stem cell matrix (SCM & SCM2) compendia

We obtained the normalised gene expression data of the stem cell matrix (SCM) compendium, consisting of 219 samples (59 deemed pluripotent, and 160 non-pluripotent) (1). Among the pluripotent samples there were 48 hESCs, 5 teratocarcinomas, 3 iPSCs and 3 germ tumour cell samples. All the SCM data were generated using Illumina Human Reference8 arrays.

In addition, we also obtained the normalised gene expression data (Illumina HT12v3 expression arrays, GSE30652) of a subset of the samples used in the SCM2 compendium, consisting of 107 hESC lines, 52 iPSC samples and 32 samples from differentiated tissue (2).

hESC, MSC and iPSC data sets

We obtained the normalised data from GEO (3) of two studies profiling human embryonic stem cell (hESC) lines and derived mesenchymal stem cells (MSC). One study (4) profiled a hESC stem cell line (H1) at 3 different passages, giving 3 replicates, as well as two MSC precursors and a bone marrow derived MSC population. These 6 samples were measured with Affymetrix HG-U133A arrays. The other study (5) profiled two hESC cell lines and MSCs derived from these two, all 4 samples done in triplicate leading to a total of 12 samples. These were generated using the Affymetrix HG-U133 Plus 2 platform.

We also downloaded the normalised data for three additional studies profiling bone-marrow derived mesenchymal stem cells (MSC-BM), all done on the same Affymetrix HG-U133 Plus 2 arrays (6-8). Samples however varied in terms of donor, donor age and passage numbers. Set1 (GSE7888) (6) consisted of a total of 23 MSC-BM samples, Set2 (GSE9593) (7) of 13 MSC-BM samples, and Set3 (GSE9520) (8) of 6 MSC-BM samples.

In addition, we downloaded the normalised data for three studies profiling hESC samples (9-11), again all done on the same Affymetrix HG-U133 Plus 2 arrays to allow comparisons between them and to the MSC-BM samples from above. The number of hESC samples per set were 5, 5 and 10 for Set1 (GSE7896) (9), Set2 (GSE13828) (10) and Set3 (GSE15148) (11), respectively. Set2 (GSE13828) also profiled induced pluripotent stem (iPS) cells from skin fibroblast samples taken from a child with spinal muscular atrophy (10). Specifically, in addition to the 5 hESC samples, there were 3 iPSC samples and 2 skin fibroblast samples. Set3 (GSE15148) contained 16 induced pluripotent stem cell lines (iPSC) derived using episomal vectors from 2 foreskin samples (11). Another data set comparing iPSCs (n=12) to parental fibroblasts (n=6), including hESCs (n=20) was obtained from (12). The raw data, generated with Affy HG HT U133A arrays, was quality checked and RMA normalised. Thus, all these data sets allowed a three-way comparison between adult diffentiated cells, the iPSCs derived from them, and hESCs.

Differentiation of Mesenchymal stem cells into osteoblasts and chondrocytes

We downloaded the normalised data for two studies (13, 14) profiling MSC samples and differentiated osteoblasts and chondrocytes, again all done on the same Affymetrix HG-U133 Plus 2 arrays to allow comparisons between them and to the hESC and MSC-BM samples from above.

Combined haematological data set

We obtained the normalised data from GEO for three studies. One study profiled five CD34+ HSC samples and five differentiated neutrophil samples (polymorphonuclear neutrophils-PMN) using the Affymetrix Human Genome U133A Plus 2 array (15). The other studies had profiled 3 and 4 CD34+ HSC cell samples using the same Affymetrix platform (16, 17), allowing direct comparison of the CD34+ HSCs. We also obtained the normalised data from the HaemAtlas as presented and used in

(18). The array for this study was the Illumina Human WG-6 v2 Expression beadchip. However, both arrays led to integrated networks of similar size encompassing effectively the same genes, allowing direct comparison.

Time course de-differentiation and re-differentiation experiment of retinal pigment epithelial (RPE) cells

The RPE dedifferentiation and redifferentiation normalised data set was obtained from ArrayExpress (E-MTAB-854), where a detailed experimental protocol can be found. Briefly, a single cell suspension of RPE was derived from hESCs and plated in 96 well plates at two densities in triplicate, a high density (100,000 cells/cm^2) and a low density (8000 cells/cm^2), and cultured for 5 weeks. RNA was extracted from the starting RPE cell suspension and from the plated cells at 8 subsequent time points (1,2,3,7,15,19,29 and 35 days after plating). In the high density plates cells proliferate and de-differentiate (acquiring a mesenchymal morphology) during the first 5 days before re-differentiating to RPE by the end of the 5 weeks. In the low density plates cells proliferate and de-differentiate for the first 7 days maintaining their mesenchymal morphology by the end of the 5 weeks. RNA was profiled on Illumina HumanHT12v4 arrays.

Time course HL60 neutrophil expression data

We obtained from GEO, the raw CEL files for the microarray (Affymetrix Human Genome U95 Version 2 Array) dataset collected by Huang et al (19) describing HL60 progenitor cells differentiating into neutrophils. The dataset consists of 25 samples; the first sample is of HL60 progenitor cells during proliferation and the remaining 24 samples correspond to two time courses, each of 12 time points (2 hrs, 4 hrs, 8 hrs, 12 hrs, 18 hrs, 1 day, 2 days, 3 days, 4 days, 5 days, 6 days, 7 days), describing differentiation of HL60 progenitors into neutrophils. Each time course corresponds to differentiation initiated via stimulation with a given medium; either DMSO or ATRA. The dataset underwent quality control assessment via the arrayQualityMetrics package in R, and was normalised using RMA.

Normal and cancer tissue, and cancer cell line data sets

We obtained from GEO the normalised data of four gene expression data sets, all using Affymetrix U133 Plus 2 arrays, profiling sufficient numbers of both normal and cancer tissue specimens. Tissues included liver (20), pancreas (21), colon (22) and stomach (23). The colon set also included colon cancer cell lines. For the other tissue types, we obtained corresponding cancer cell lines, profiled on the same Affymetrix arrays, from the Cancer Cell Line Encyclopedia (24).

Normal and cancer stem cell data sets

We collected the normalised data from GEO of two neural and gioma stem cell gene expression data sets, both using Affymetrix U133 Plus 2 arrays. One data sets profiled a neural stem cell line (in duplicate) plus replicates from 4 different glioma stem cell lines (Materials and Methods, (25)). The second data set profiled normal human fetal (hf) neural stem cells and tumorigenic glioma neural stem cell lines, which had been expanded using growth factors EGF and FGF in adherent culture conditions (26). Under these conditions apoptosis and differentiation are suppressed resulting in more homogeneous populations of stem cells (26).

We collected the normalised data from GEO of 12 hematopoietic and 12 leukemic stem cell (LSC) samples (27). The LSCs were from CD34+ chronic myelogenous leukemia patients. All samples were profiled on Affymetrix U133 Plus 2 arrays.

Cancer stem cell and parental cancer cell data set

In order to compare putative cancer stem cells (CSC) to their parental tumour cell (PTC) lines we used the normalised data (GEO) from (28). Specifically, we focused on five tissues: breast with 2 CSCs and 3 PTCs, brain with 5 CSCs and 4 PTCs, lung with 5 CSCs and 3 PTCs, oral cavity with 8 CSCs and 5 PTCs and colon with 7 CSCs and 4 PTCs. In the case of colon we used positivity of CD133, a colon stem cell marker to assign CSC/PTC status (samples on GEO are likely to have been mislabeled). Reported results across all tissue types is however independent of inclusion or exclusion of the colon subset. All samples were profiled on Affymetrix HG-U133 Plus2 arrays.

Pluripotency signature(s) and the TPSC pluripotency score

We considered two pluripotency gene expression signatures: (1) A 19-gene pluripotency signature, consisting of 11 up- and 8 down regulated genes in pluripotent cells (29). For this signature, the pluripotency score of a given sample was derived as the t-statistic comparing the expression levels of the upregulated genes to the downregulated ones. We call this score construction method, the t-statistic based pluripotency score, abbreviated as TPSC. (2) A 189-gene pluripotency expression signature derived in Palmer et al (30). This signature only consists of a list of genes and is not a single sample predictor. To construct one, we followed the principal component analysis (PCA) procedure outlined in (30). Thus, we performed a focused PCA on the stem cell matrix (SCM) compendium on the genes from the signature present in that array (a total of 157 genes). The top PC from the PCA correlated with pluripotency status (Wilcoxon rank sum, $P<10^{-10}$), thus validating the signature in the SCM data. Thus, a pluripotency score of an independent sample can be obtained by correlation of the loadings of the top principal component with the sample's gene expression profile. Because this method is sensitive to the normalisation strategy, we also constructed an alternative score based on the t-test procedure. Specifically, we used the sign of the loadings in the top principal components to assign the 157 genes into up and downregulated categories, having fixed the overall sign of the principal component also from the SCM data. By comparing, in a given sample, the expression levels of the up and down regulated genes using a t-test we obtain the sample-specific TPSC score.

Protein Interaction Network

We downloaded the full Protein Interaction Network (PIN) from Pathway Commons (www.pathwaycommons.org) (31) (date stamp 13th June 2012). Using this comprehensive resource, we built an integrated network including the Human Protein Reference Database (32), the National Cancer Institute Nature Pathway Interaction Database (NCI-PID) (*pid.nci.nih.gov*), the Interactome (Intact) *http://www.ebi.ac.uk/intact/* and the Molecular Interaction Database (MINT) *http://mint.bio.uniroma2.it/mint/*. Protein interactions in this network include physical stable interactions such as those defining protein complexes, as well as transient interactions such as post-translational modifications and enzymatic reactions found in signal transduction pathways, including 20 highly curated immune and cancer signaling pathways from NetPath (*www.netpath.org*) (33). Redundant interactions were removed and only genes with an Entrez gene ID annotation were retained.

To remove network edges which may likely represent false positives, we adopted a sparsification procedure based on imposing a signaling hierarchy (albeit bi-directional) on the network. This procedure removes edges between proteins whose main cellular localisations are not adjacent in the context of the layers defining the signaling hierarchy. Imposing a signaling hierarchy on a PIN is a procedure which has already been successfully used in other contexts (34). Briefly, each node (protein) in the PIN is assigned a main cellular localisation ("a signaling layer") by using the Gene Ontology annotation (34). Specifically, we first selected all Entrez ID genes annotated to the extracellular space/region using the following set of GO-terms: GO:0005102, GO:0008083, GO:0005125, GO:0005615, GO:0005576, GO:0044421. Next, we identified all Entrez ID genes annotated to transmembrane receptor activity (GO:0004888, GO:0004872, GO:0005886), and finally all genes annotated to the intracellular domain or to biological functions associated with the intracellular region (GO:0005622, GO:0044424, GO:0005634, GO:0005737, GO:0005829, GO:0000139, GO:0035556, GO:0007243, GO:0006468). This included GO terms such as cell-nucleus, cytoplasm, cytosol, golgi membrane, intracellular signal transduction, kinase cascade, protein phosphorylation. Because genes may be annotated to multiple domains, we constructed mutually exclusive sets by favouring the more "external" domain, so that a gene annotated to both extracellular and transmembrane domains was allocated to the extracellular domain only, and a gene annotated to both transmembrane and intracellular domains was assigned to the transmembrane domain only. Thus, all Entrez ID genes were annotated to one of the extracellular ("EC"), transmembrane receptor ("MR") or intracellular ("IC") domains. Next, we selected those nodes in the PIN which could be assigned to one of these three domains. The resulting PIN was then pruned by removing edges (interactions) which were not consistent with the signaling hierarchy structure. Thus, only edges with corresponding end nodes in the following combinations were allowed: EC-EC, EC-MR (or MR-EC), MR-IC (or IC-MR) and IC-IC. This resulted in a maximally connected PIN of 8,434 nodes and over 300,000 interactions.

Integration of PIN with gene expression data

To integrate the gene expression data with the PIN, expression profiles of probesets mapping to the same Entrez gene identifier in the PIN were averaged. Proteins in the PIN without probesets representing their coding genes on the array, were removed from the PIN. The resulting maximally connected component of the PIN defined a sparse network with the number of nodes (genes) depending on the arrays used. In the case of Affymetrix U133 Plus 2 arrays (the most widely used array in this work), the resulting maximally connected integrated PIN consisted of 8290 nodes and 299459 edges. For the Affymetrix Human Genome U95 v2 arrays, the integrated PIN consisted of 5555 nodes and 175640 interactions. For the Affymetric U133A arrays, the PIN was of size 7027 nodes and 246005 edges. Finally, for the Illumina Human WG-6 v2 expression beadchip, the integrated maximally connected PIN consisted of 8135 nodes and 290360 edges, i.e very similar in size to the Affymetrix U133 Plus 2 arrays.

Gene Set Enrichment Analysis (GSEA)

Functional annotation and GSEA was performed using the DAVID Bioinformatics Resources 6.7 (35). The proteins in the PIN were used as a background gene set and P-value estimated using Fisher's exact test. Adjusted P-values used the Benjamini-Hochberg correction as implemented in DAVID.

Construction of the sample specific stochastic matrix and network entropy rate

In this work we invoke the mass action principle in order to be able to define a stochastic matrix for each individual sample. In detail, let E_i denote the normalised expression level of gene i in a given sample. For a given neighbour $j \in N(i)$ (where $N(i)$ labels the neighbours of i in the PIN), the mass-action principle states that the probability of interaction with i is approximated by the product $E_i E_j$. Normalising this to ensure that $\sum_j p_{ij}=1$, we get

$$p_{ij}=\frac{E_{js}}{\sum_{k \in N(i)} E_{ks}} \quad \forall j \in N(i) \qquad \text{(equation 1)}$$

Clearly, if $j \notin N(i)$, then $p_{ij}=0$. This then defines a sample-specific stochastic matrix. From this stochastic matrix one can then construct a local network entropy for each gene i in the PIN, as

$$S_i=-\sum_{j \in N(i)} p_{ij} \log p_{ij}$$

which reflects the level of uncertainty or redundancy in the local interaction probabilities around gene i. We note that the above expression for the local entropy is not normalised so that the maximum possible entropy depends on the degree (k_i) of the node i. In fact,

$$\max S_i=\log k_i$$

Thus, it is convenient to also define a normalised local entropy as (see (36)),

$$\tilde{S}_i=-\frac{1}{\log k_i}\sum_{j \in N(i)} p_{ij} \log p_{ij}$$

We stress again that this local network entropy can be computed for each gene i in each given sample.

When defining a global network entropy (i.e. for the whole network) one can, in principle, consider the average of these normalised local entropies. This average however is a non-equilibrium entropy, in contrast to the global entropy rate, S_R, which is defined in terms of the stationary distribution, π, of the stochastic matrix p, i.e. through $\pi p=\pi$. Specifically, the global entropy rate, S_R, is defined by (37)

$$S_R = \sum_i \pi_i S_i$$

where S_i are the unnormalised local entropies.

We note that the network entropy rate is bounded between 0 and a positive maximum value that depends only on the adjacency matrix of the network (38). Indeed, it can be shown that the maximum possible entropy rate is attained by a stochastic matrix, p_{ij}, defined by

$$p_{ij} = \frac{A_{ij} v_j}{\lambda v_i}$$

where A_{ij} is the adjacency matrix (i.e. unweighted) of the PIN, and v and λ are the dominant eigenvector and eigenvalue of this adjacency matrix, respectively. The maximum attainable entropy rate, M_R, will thus depend on the specifics of the network, including total number of nodes and edges. Thus, if desired, the network entropy rate, S_R, can be scaled relative to the maximum attainable value, $\tilde{S}_R \equiv S_R / M_R$, so that $\tilde{S}_R$ is bounded between 0 and 1.

We also note that there is an alternative construction of the stochastic matrix. In fact, the construction of the local stochastic matrix around a given gene *i* given above does not depend on the expression level of gene *i*. This means that a gene can have a low network entropy despite the fact that it is not expressed. Since this may not be desirable we also consider an alternative construction of the network entropy which "enforces" a high local network entropy for genes that are not expressed. This is achieved by replacing p_{ij} in the equation above with

$$p_{ij} = \frac{1-\beta(E_{is})}{k_i} + \beta(E_{is}) \frac{E_{js}}{\sum_{k \in N(i)} E_{ks}} \quad \forall j \in N(i) \qquad \text{(equation 2)}$$

where β is a function of the gene *i*'s expression value, E_{is}, taking values on the compact support [0,1]. We define the function $\beta(E_{is})$ as follows: $\beta(E_{is})=1$ if and only if $E_{is}>\alpha_h$, $\beta(E_{is})=0$ if and only if $E_{is}<\alpha_l$ and $\beta(E_{is})=(E_{is}-\alpha_l)/(\alpha_h-\alpha_l)$ for all $\alpha_l \leq E_{is} \leq \alpha_h$. We choose α_l and α_h as the 10th and 90th quantile of the sample's genomewide expression profile. In this work we report the S_R values obtained from the construction in equation 1 above. However, using the alternative construction (equation 2) leads to similar results.

Simulation and entropy rate perturbation analysis

As a proof of concept that network entropy provides a measure of the degree of signalling promiscuity in a network, we performed a simulation study. Without loss of generality, we randomly sampled 2000 genes from the complete integrated PIN (~7800 nodes) obtained by integrating the gene expression data from the SCM with our constructed PIN. This resulted in a maximally connected component consisting of ~1500 genes. This reduced network size allowed faster computation of the network entropy as required for our simulation study. To simulate a ground state, representing a pluripotent poised state, the corresponding stochastic matrix was defined as one where the associated random walk is unbiased, i.e. $p_{ij} = 1/k_i$, whenever $j \in N(i)$, 0 otherwise. Next, we considered two types of perturbation. In one case, we picked all nodes of degree>=2 in the network, and for each such node, a randomly picked edge was then assigned a large weight (in the range 0.8-0.95), with the rest of edges assigned a low weight (~0.1), ensuring that the sum of weights equals 1, as required for a stochastic matrix. This was done for each node in the network separately, resulting in >1000 perturbations and associated entropy rates. In the second perturbation analysis, we generated whole signal transduction pathways, in which a specific path was generated at random, which the weights of the visited nodes in the path modified as described earlier. Path lengths were chosen to be of maximum length 9, corresponding to the diameter of the network, although generated paths often led to shorter paths (since a requirement was not to visit the same node twice). In this perturbation analysis, we generated 100 distinct activation pathways and computed the resulting entropy rates. Thus, by using these two types of perturbation and comparing the entropy rates before and after the perturbation, we can assess the impact of single gene perturbations (activation of single genes) and activation of complete pathways.

Supplementary Figures:

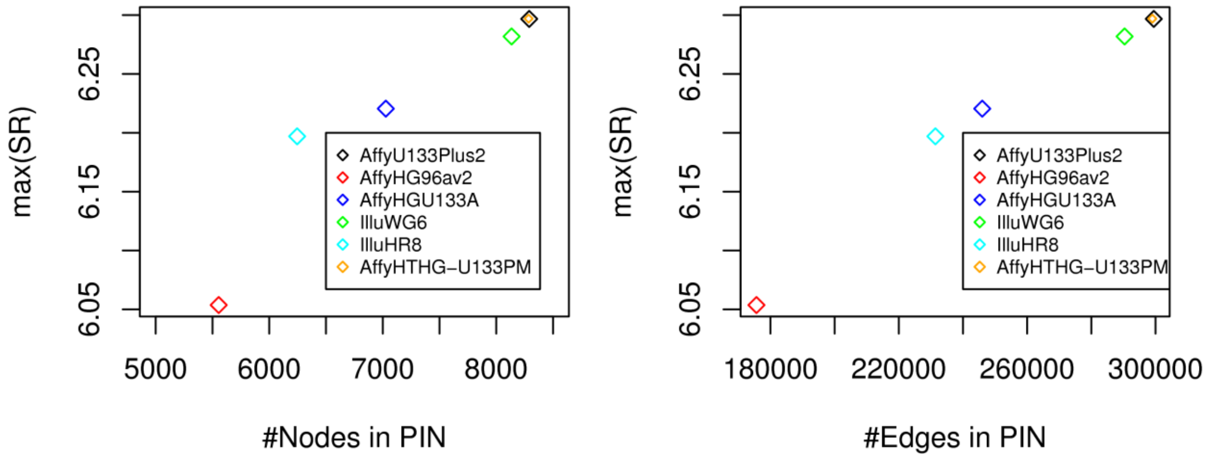

Supplementary Figure S1: Scatterplots of the maximum possible entropy rate (maxSR) against the number of nodes (left panel) and edges (right panel) in the respective integrated PINs for six different arrays considered in this work. Observe how the maxSR scales with the size of the resulting network. We note that the integrated networks from the different arrays may differ in the precise genes represented in them, hence why the network topology might be slightly different, and hence why deviations from a linear relation may be expected.

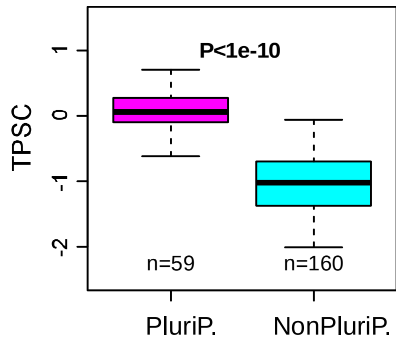

Supplementary Figure S2: The pluripotency score (TPSC) of the 19-gene pluripotency signature (29) between the 59 pluripotent and 160 non-pluripotent cell-lines from the stem cell matrix (SCM) compendium (219 samples). P-value is from a Wilcoxon rank sum test.

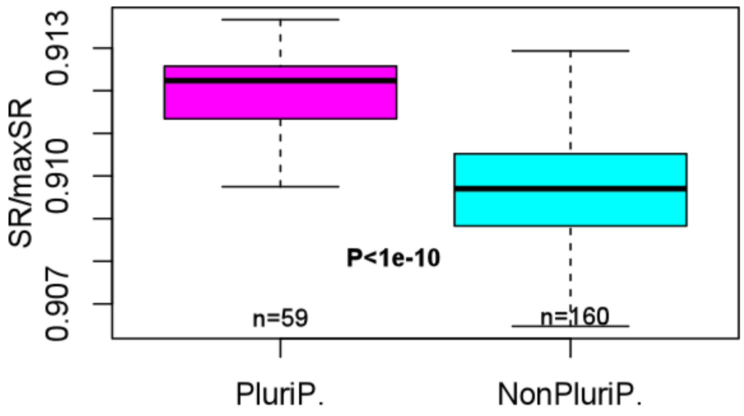

Supplementary Figure S3: Comparison of the normalised entropy rates in the SCM compendium comparing pluripotent to non-pluripotent cell types, where the entropy rate (SR) was computed after removing cell-proliferation and cell-cycling genes defined in Ben-Porath et al (39). Wilcoxon rank sum test P-value is given.

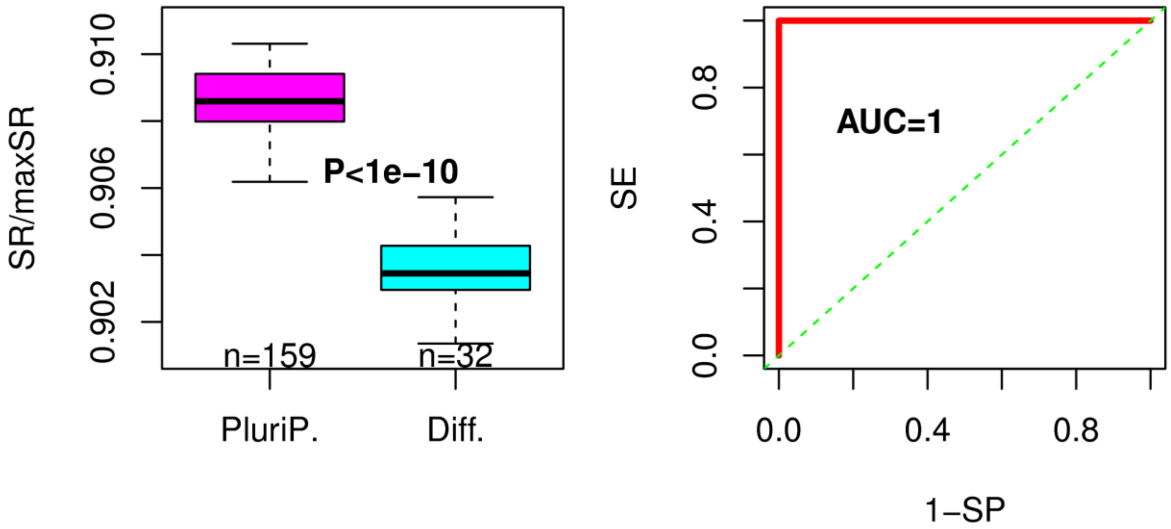

Supplementary Figure S4: Comparison of the normalised entropy rates in the SCM2 compendium comparing pluripotent to differentiated cell types, where the entropy rate (SR) was computed after removing cell-proliferation and cell-cycling genes defined in Ben-Porath et al (39). Wilcoxon rank sum test P-value is given. Right panel shows the corresponding ROC curve and AUC.

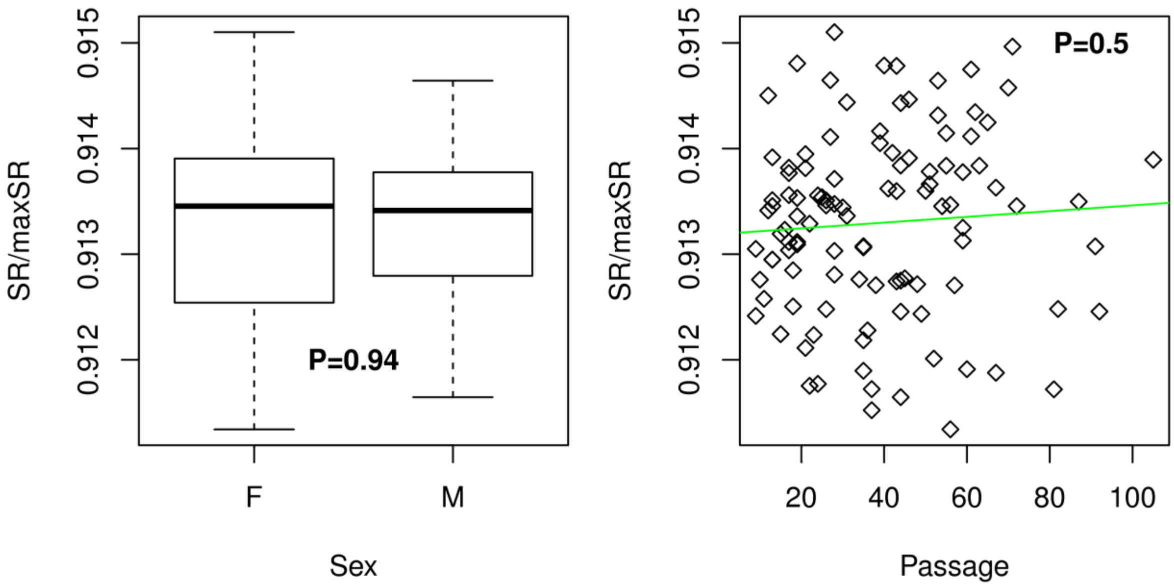

Supplementary Figure S5: Comparison of the normalised entropy rates of 107 hESCs from the SCM2 compendium (2) according to sex (left panel) and passage number (right panel). Wilcoxon rank sum test P-value is given in left panel. Linear regression t-test P-value is given in the right panel.

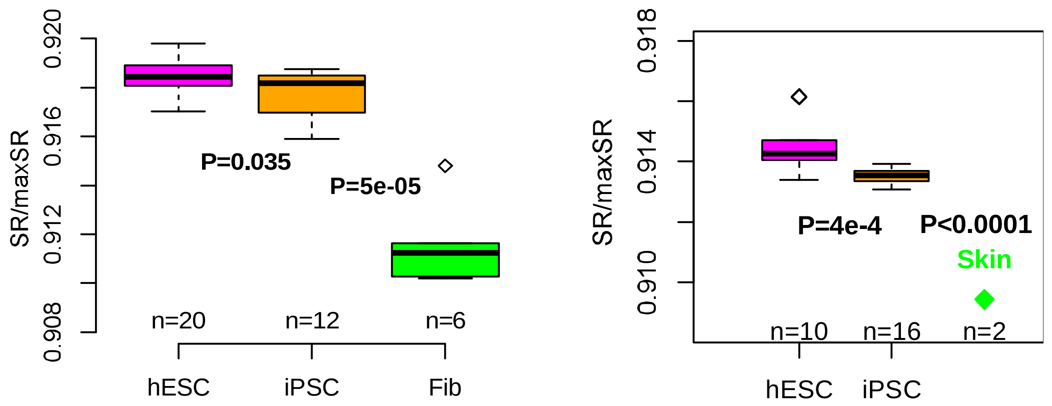

Supplementary Figure S6: Left panel: comparison of normalised network entropy values of fibroblasts (green), their induced pluripotent cells (iPSCs, orange) and hESC controls (magenta). All samples done on same arrays as part of the same study (12). Right panel: comparison of normalised network entropy values of two adult differentiated foreskin samples (green), their induced pluripotent cells (iPSCs, orange), and hESC controls (magenta). All samples done on same arrays as part of the same study (11). Wilcoxon rank sum test P-values between the respective groups are given, as indicated, except for the comparison between iPSCs and the two skin samples in the right panel for which a t-test was used.

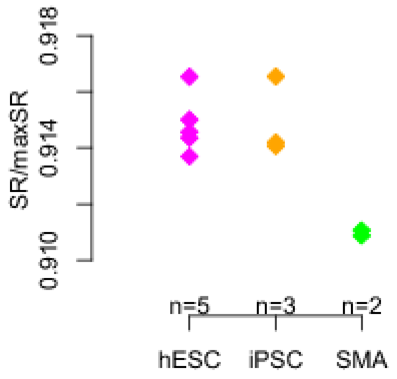

Supplementary Figure S7: Comparison of normalised network entropy values of two differentiated spinal muscular atrophy samples (green), induced pluripotent cells (iPSCs, orange) derived from these, and hESC controls (magenta). Expression data is from study Ebert et al (10) , which used Affymetrix HG-U133 Plus2 arrays for all samples.

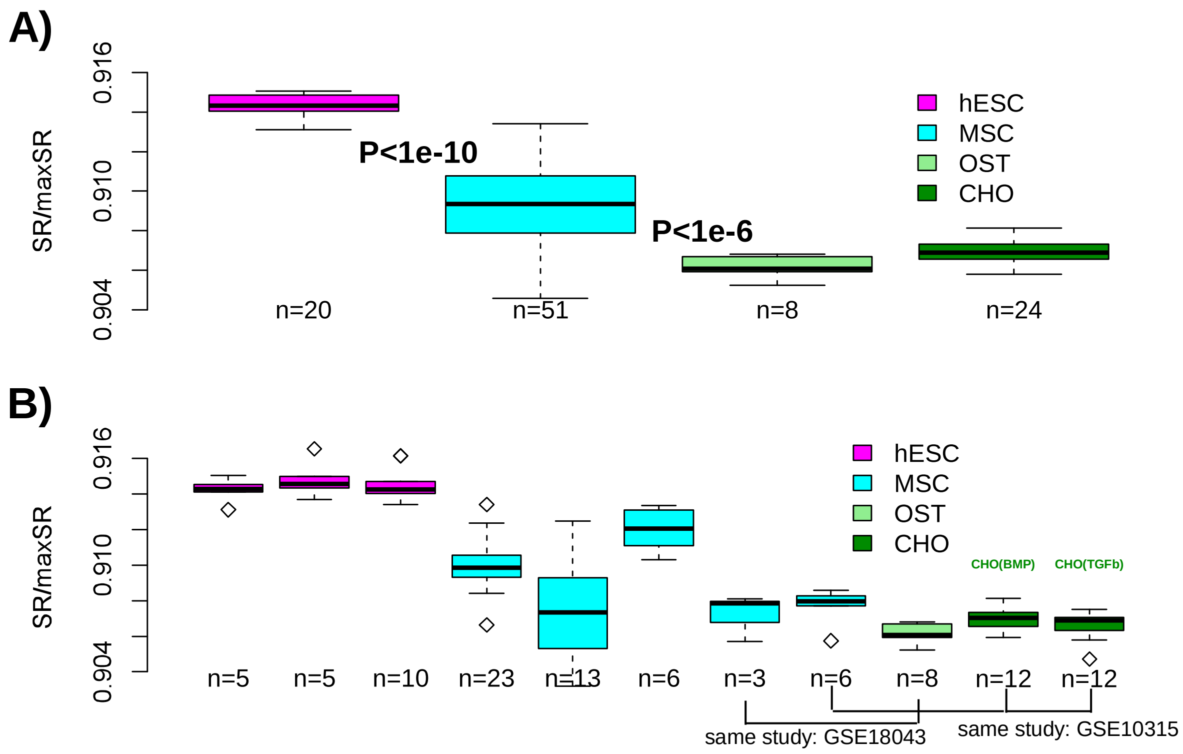

Supplementary Figure S8: Network entropy in the mesenchymal lineage: A) Comparison of normalised network entropy values of hESCs, bone marrow MSCs (MSC-BM), differentiated osteoblasts and chondrocytes. (see Supplementary Materials for data sets used), as indicated. All samples were generated using the same Affymetrix U133 Plus2 platform. Wilcoxon rank sum test P-value between all hESCs and all bone marrow MSCs is given, as well as between all MSCs and the combined osteoblasts and chondrocytes. **B)** As A), but now samples have been broken up into the different studies from which they were derived, demonstrating the relative robustness of network entropy across studies profiling the same cell-type, although MSCs exhibited some variation. Note that 3 MSC samples and 8 osteoblasts were done as part of the same study (GSE18043), whereas also 6 MSCs and 24 chondrocytes were done as part of the same experiment (GSE10315). Specifically, in GSE10315, MSCs were induced to differentiate into chondrocytes with either BMP2 or TGFb.

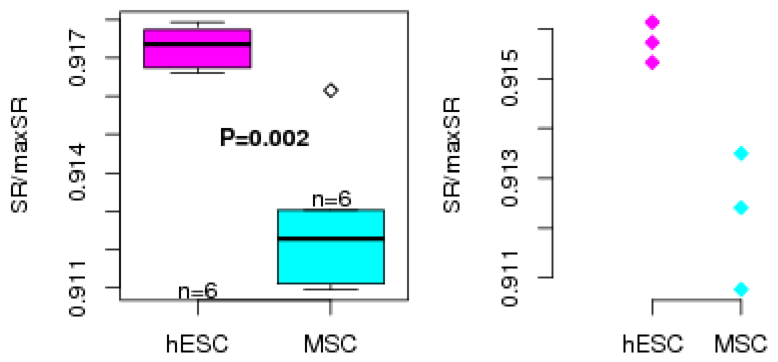

Supplementary Figure S9: Normalised network entropy rates of human embryonic stem cells (hESCs) and mesenchymal stem cells (MSCs) derived from these. In left panel, samples were profiled with the Affymetrix HG-133U Plus 2 arrays and were taken from Giraud-Triboult et al (5), whilst those on the right were obtained from Barberi et al (4) and were generated with Affymetrix HG-U133A arrays. In left panel we provide the Wilcoxon rank sum test P-value.

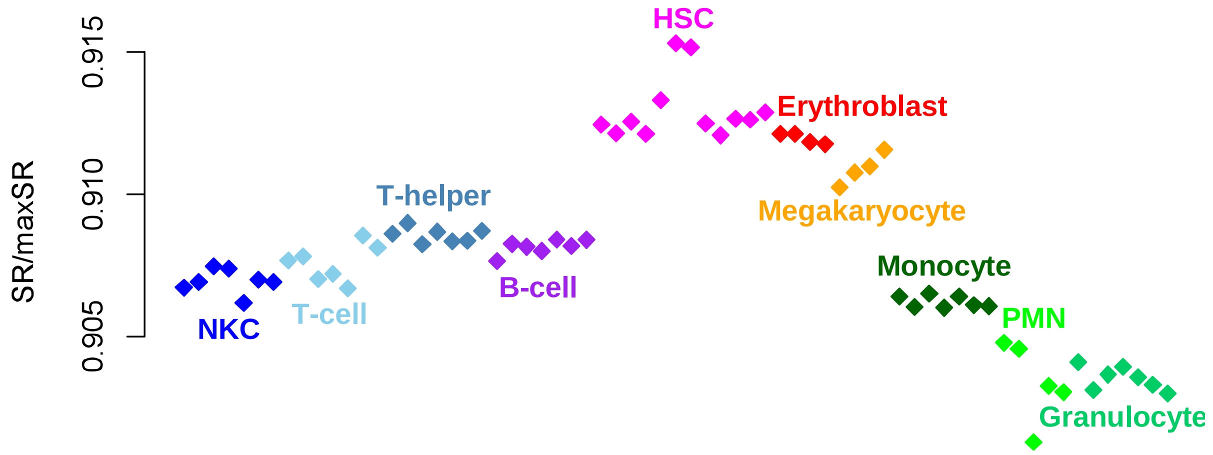

Supplementary Figure S10: Comparison of the network entropy rates for major blood cell types in the hematopoietic system, as indicated. Blood cell types have been arranged with lymphoid cells to the left, and myeloid (monocytes & granulocytes) cells to the right, with the pluripotent HSCs and less differentiated erythroblasts/megakaryocytes in the middle. Observe how the network entropy is lower for the more differentiated lymphoid and myeloid lineages. The HSCs and PMNs (polymorpho neutrophils) were profiled with the Affymetrix U133 Plus 2 platform, while the rest of samples were profiled with Illumina Human WG6v2 arrays. See Supplementary Methods for the “Combined haematological data set”. Observe how the PMNs done on the Affymetrix array are closest to the granulocytes done on the Illumina array, consistent with PMNs being closest to granulocytes.

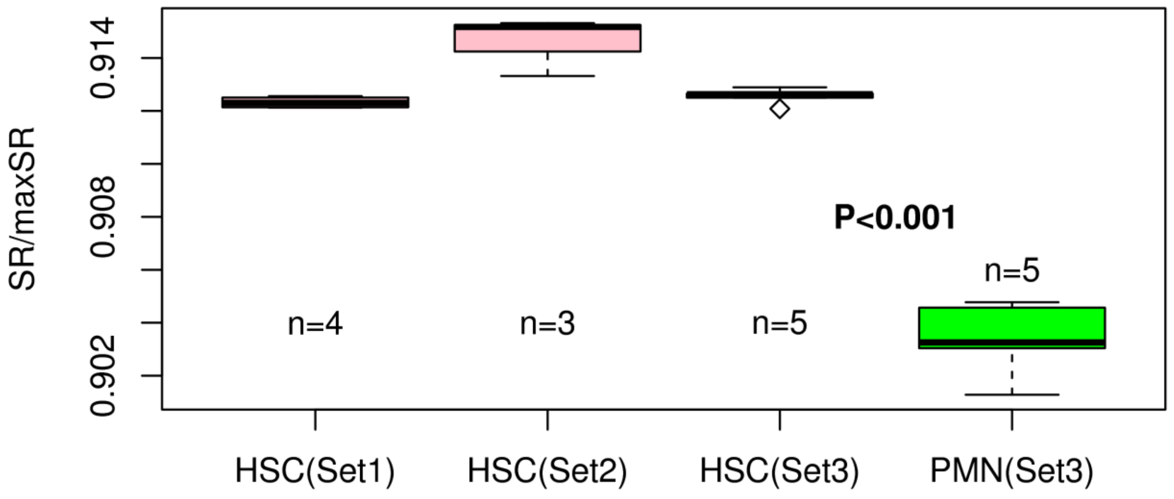

Supplementary Figure S11: Comparison of network entropy of CD34+ hematopoietic stem cells (HSCs) from three different studies (indicated as Set1, Set2 and Set3) and that of polymorphonuclear (differentiated) neutrophils (PMNs) (15) (16, 17). We note that Set3 (15) included 5 HSC and 5 PMN samples, as indicated. All 17 samples were profiled on the same Affymetrix HG-133U Plus 2 chip. Wilcoxon rank sum test P-value between CD34+ HSCs and PMNs is given.

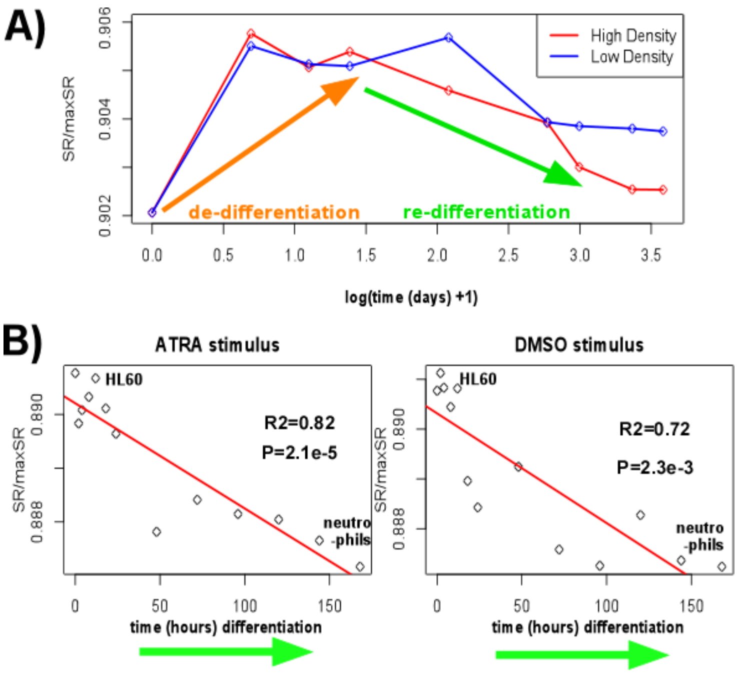

Supplementary Figure S12: Dynamic changes in network entropy after removing genes implicated in cell proliferation and cell-cyle. A) Dynamic network entropy changes in a time course de-differentiation and re-differentiation experiment of retinal pigment epithelium (RPE), with cell density indicating the initial plating density of RPE cells (Supplementary Materials). **B)** Plots of normalised network entropy rate (SR/maxSR, y-axis) of HL60 leukemic progenitor cells against time from initial stimulus with either ATRA or DMSO. The data points on the left indicate the less differentiated HL60 cells, whereas the ones on the far right represent

differentiated neutrophils. All samples were profiled on the same Affymetrix HG95v2 Array. We provide the R^2 values and associated P-values from a linear regression. In both A) and B), the network entropy was computed after removing the cell-proliferation and cell-cycling genes of Ben-Porath et al (39).

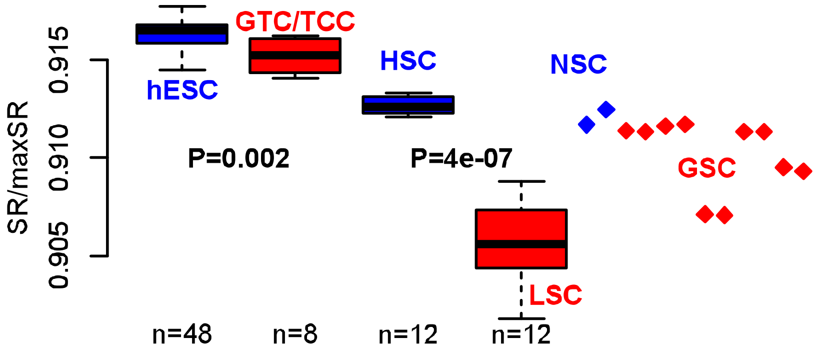

Supplementary Figure S13: Normalised network entropy rates of human embryonic stem cells (hESCs,n=48) and combined teratocarcinoma (n=5) / germ tumour (n=3) cell lines from the stem cell matrix compendium (all deemed pluripotent), as well as of 12 hematopoietic stem cells (HSC) and 12 CD34+ chronic myeloid leukemic stem cells (LSC) (Supplementary Materials). Wilcoxon rank sum test P-value between the pluripotent normal (hESC) and cancer (GTC/TCC) cells is given, as well as between the HSCs and LSCs. Finally, also included are the normalised network entropy rates (SR/maxSR) of a neural stem cell line (NSC) (in duplicate, blue) compared to that of four different MGG glioma stem cells (red, with replicates) from (25), as indicated.

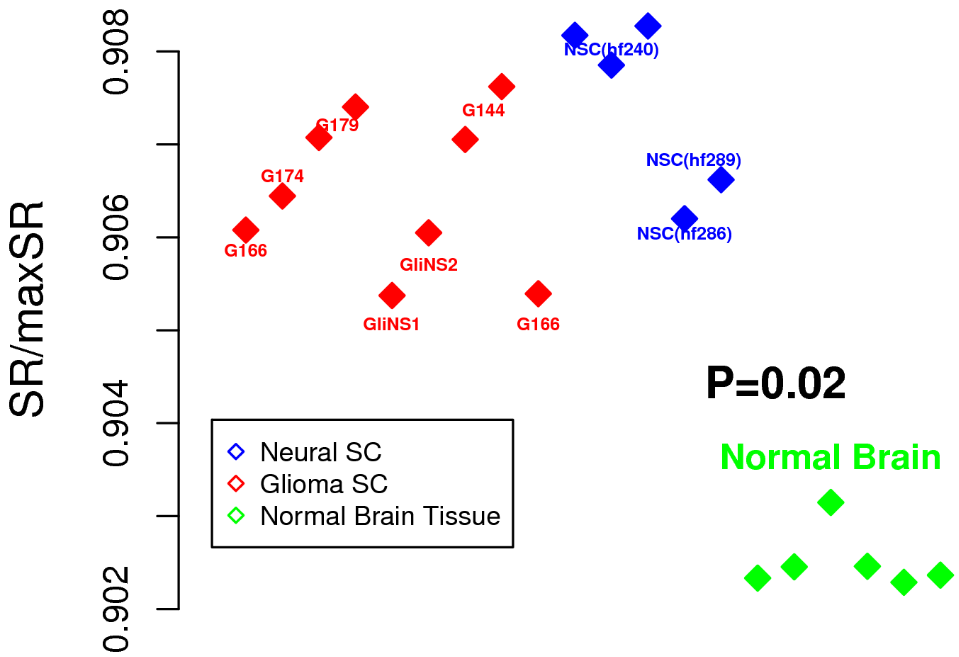

Supplementary Figure S14: Normalised network entropy rates of a set of glioma (red) and neural stem cell (blue, NSC) lines (with replicates), plus samples from normal differentiated brain cortex (green). Expression data is from Pollard et al (26) and was generated using Affymetrix HG-U133 Plus 2 arrays. P-value is from a Wilcoxon rank sum test comparing the 3 different NSCs to the 6 independent normal brain samples.

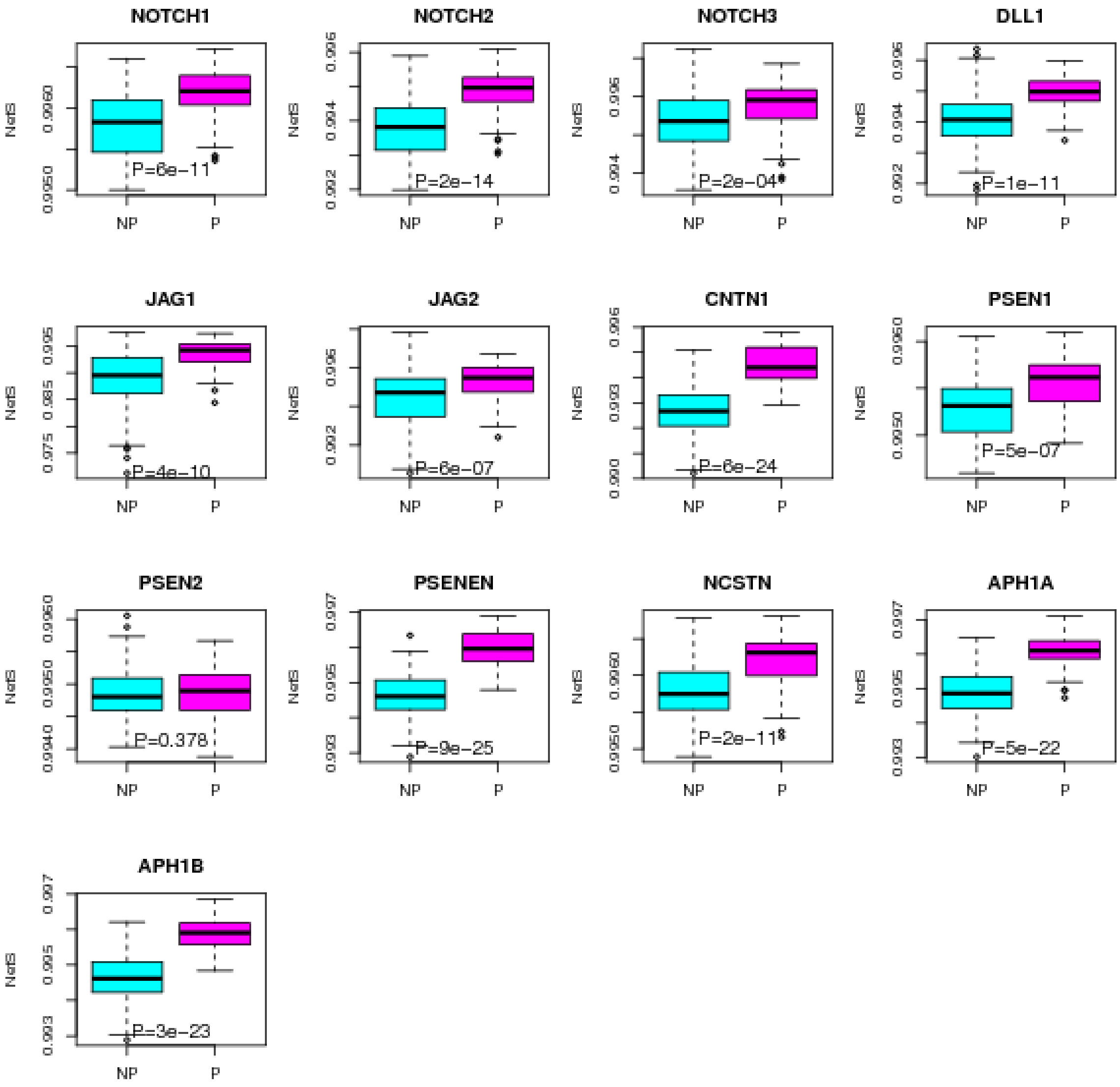

Supplementary Figure S15: Comparison of local network entropy values (y-axis) of the 13 Notch pathway components between the 59 pluripotent and 160 non-pluripotent samples of the stem cell matrix compendium. Wilcoxon rank sum test P-values are given.

LOCAL NETWORK ENTROPY LOSSES PREDICTS ACTIVATION OF NOTCH SIGNALING IN NON-PLURIPOTENT CELLS

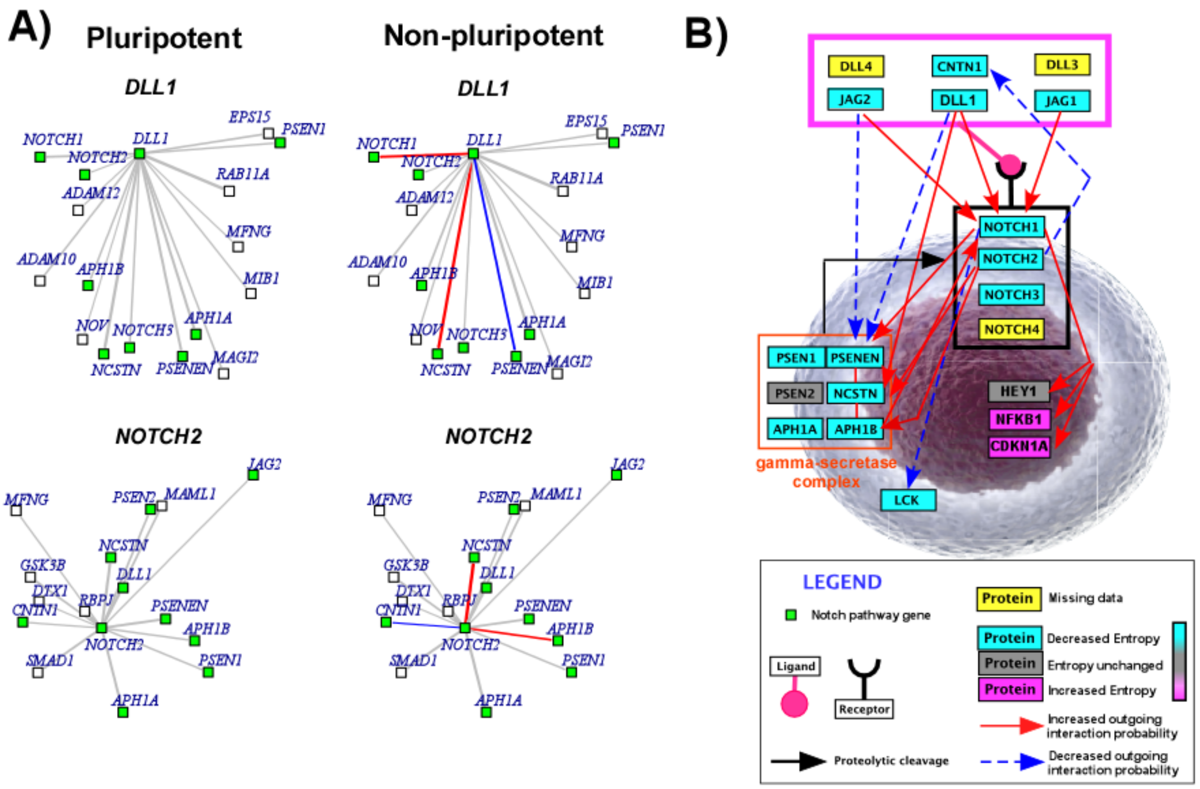

Supplementary Figure S16: Local network entropy analysis identifies activation of Notch signaling in the non-pluripotent state: A) Interaction probability distributions around two Notch pathway genes (*DLL1* and *NOTCH2*) in the pluripotent and non-pluripotent states. The interaction probabilities are indicated by the width of the grey edges (left panels), and were estimated as averages over the 59 and 160 pluripotent and non-pluripotent samples from the stem cell matrix, respectively. In the right panels, we indicate in color those interaction probabilities that change the most (red: increased probability, blue: decreased probability) between the pluripotent and non-pluripotent states, thus driving a lower entropy in the non-pluripotent state. **B)** Graphical rewiring diagram of the main Notch pathway components, with the main increased and decreased probability interactions depicted. Observe how most of the Notch pathway components have reduced local entropies, highlighting the fact that the interactions distribution becomes more focused (mostly increased interactions between Notch pathway members).

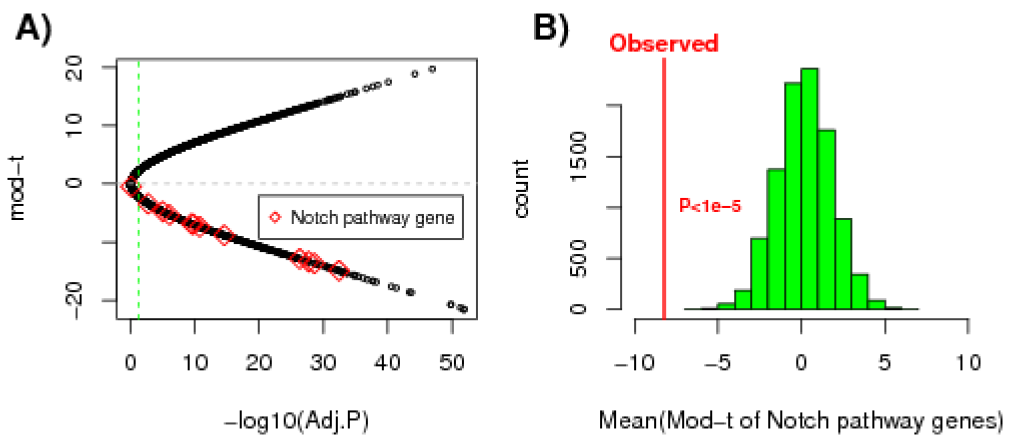

Supplementary Figure S17: A) Scatterplot of limma moderated t-statistics of local differential entropy changes against -log10(adjusted P-value), where adjustment has been done for multiple testing. **B)** Histogram of the average moderated t-statistics for randomly selected genes in the network (10000 random selections). Observed average moderated t-statistic value for Notch pathway genes shown is indicated by red line.

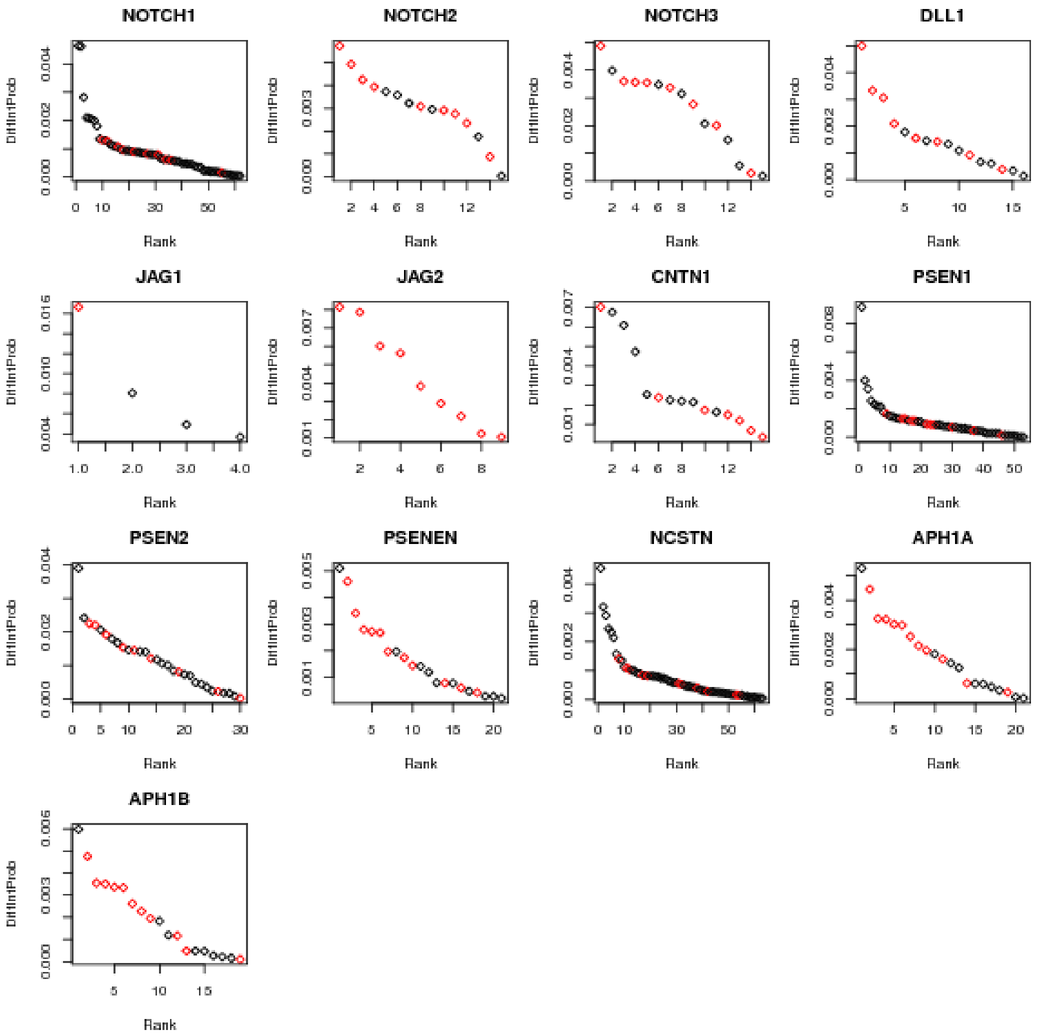

Supplementary Figure S18: For each of the 13 Notch pathway genes present in the integrated network, we plot the absolute difference in the relative interaction probability (between non-pluripotent and pluripotent states) of the corresponding Notch pathway gene with putative interactors. Those interactors which are components of the Notch signaling pathway are indicated in red.

Local Reduced Network Entropy			
GO ID	GO Term	P Value	Adjusted P value
GO:0042517	positive regulation of tyrosine phosphorylation of Stat3 protein	6.72E-07	0.0010
GO:0050731	positive regulation of peptidyl-tyrosine phosphorylation	1.65E-06	0.0013
GO:0004896	cytokine receptor activity	4.70E-06	0.0013
GO:0042516	regulation of tyrosine phosphorylation of Stat3 protein	5.20E-06	0.0026
GO:0042531	positive regulation of tyrosine phosphorylation of STAT protein	6.68E-06	0.0025
GO:0019838	growth factor binding	1.16E-05	0.0016
GO:0046427	positive regulation of JAK-STAT cascade	1.28E-05	0.0039
GO:0042509	regulation of tyrosine phosphorylation of STAT protein	2.97E-05	0.0075
GO:0002673	regulation of acute inflammatory response	3.10E-05	0.0067

Regression Model			
GO ID	GO Term	P Value	Adjusted P value
GO:0003676	nucleic acid binding	1.08E-06	0.00833
GO:0016070	RNA metabolic process	5.52E-06	0.0129
GO:0044446	intracellular organelle part	7.10E-06	0.0129
GO:0044422	organelle part	7.24E-06	0.0129
GO:0044428	Nuclear part	8.41E-06	0.0129
GO:0006350	transcription	3.18E-06	0.0407
GO:0032774	RNA biosynthetic process	5.46E-06	0.046
GO:0032991	macromolecular complex	5.60E-06	0.046
GO:0006694	steroid biosynthetic process	5.74E-06	0.046

Increased gene expression			
GO ID	GO Term	P Value	Adjusted P value
GO:0005773	vacuole	1.67E-06	2.30E-04
GO:0005764	lysosome	9.66E-07	2.66E-04
GO:0000323	lytic vacuole	9.66E-07	2.66E-04
GO:0044437	vacuolar part	5.78E-04	0.052
GO:0006952	defense response	5.59E-05	0.089
GO:0006955	immune response	2.47E-04	0.19
GO:0005886	plasma membrane	0.0035	0.21
GO:0043020	NADPH oxidase complex	0.011	0.38
GO:0015629	actin cytoskeleton	0.0089	0.39
GO:0005774	vacuolar membrane	0.013	0.41

Supplementary Figure (Table) S19: Gene Set Enrichment Analysis of (a) genes showing the most significant reductions in local network entropy as a function of differentiation stage in the HL60 time course data (19) (224 genes, top table), (b) genes selected using the regression method of Mar et al (1428 genes, middle table) (40), and (c) genes showing significantly increased expression with differentiation stage (224 genes, lower table). We list the most highly enriched gene ontology IDs, their terms, P-values of enrichment and associated Benjamini-Hochberg adjusted P-values.

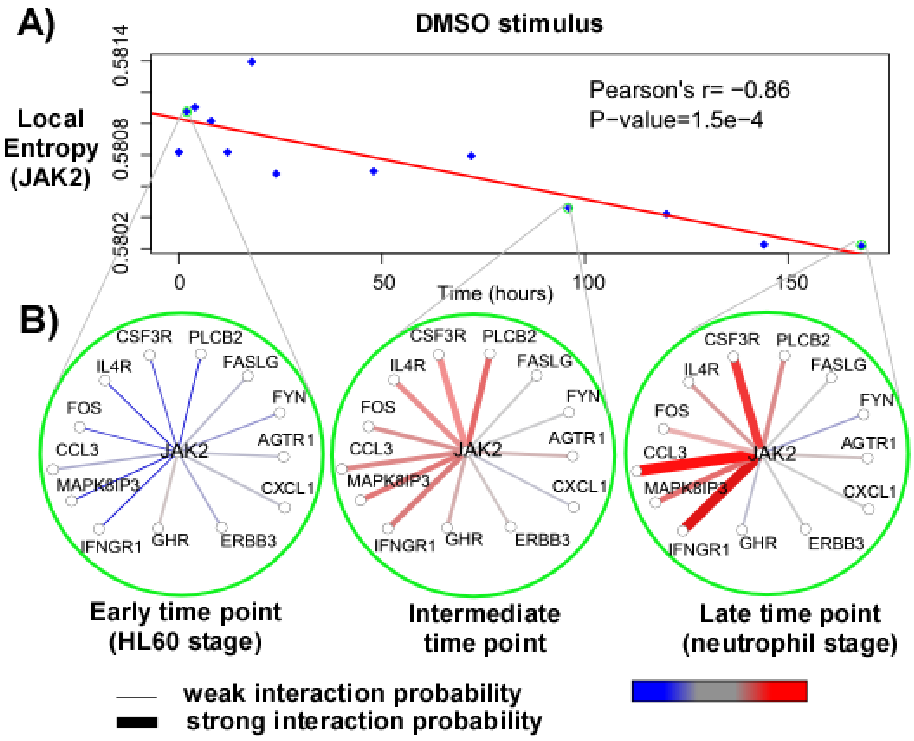

Supplementary Figure S20: Local network entropy analysis identifies JAK2 as a key player in the differentiation of HL60 cells into neutrophils: **A)** Plot of the local network entropy around JAK2 (y-axis) against time from the initial DMSO stimulus. The data points on the left indicate the less differentiated HL60 cells, whereas the ones on the far right represent differentiated neutrophils. We give Pearson's correlation coefficient (r) and associated P-value. **B)** Representative interaction network around JAK2 illustrating the reduction in the uncertainty (i.e. network entropy) of JAK2's interactions, whilst HL60 cells undergo differentiation into neutrophils.

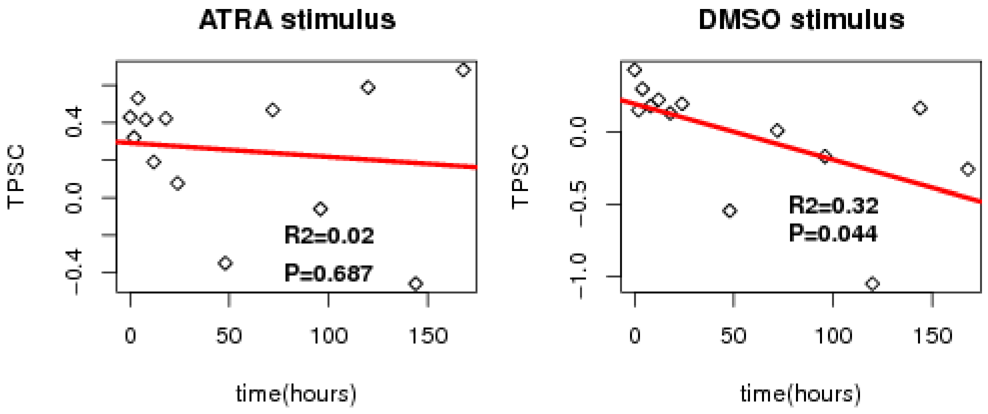

Supplementary Figure S21: Plots of the pluripotency score (derived from the Mikkelsen et al pluripotency signature (29)) (TPSC, y-axis) of leukemic HL60 cells against time from initial stimulus with either ATRA (left panel) or DMSO (right panel) stimulus. Within each panel, the data points on the extreme left indicate the less differentiated HL60 cells, whereas the ones on the far right represent differentiated neutrophils. All samples were profiled on the same Affymetrix HG95v2 array. Original expression data is from Huang et al (19) and was made available in Mar et al (40). We provide the R^2 values and associated P-values from a linear regression.

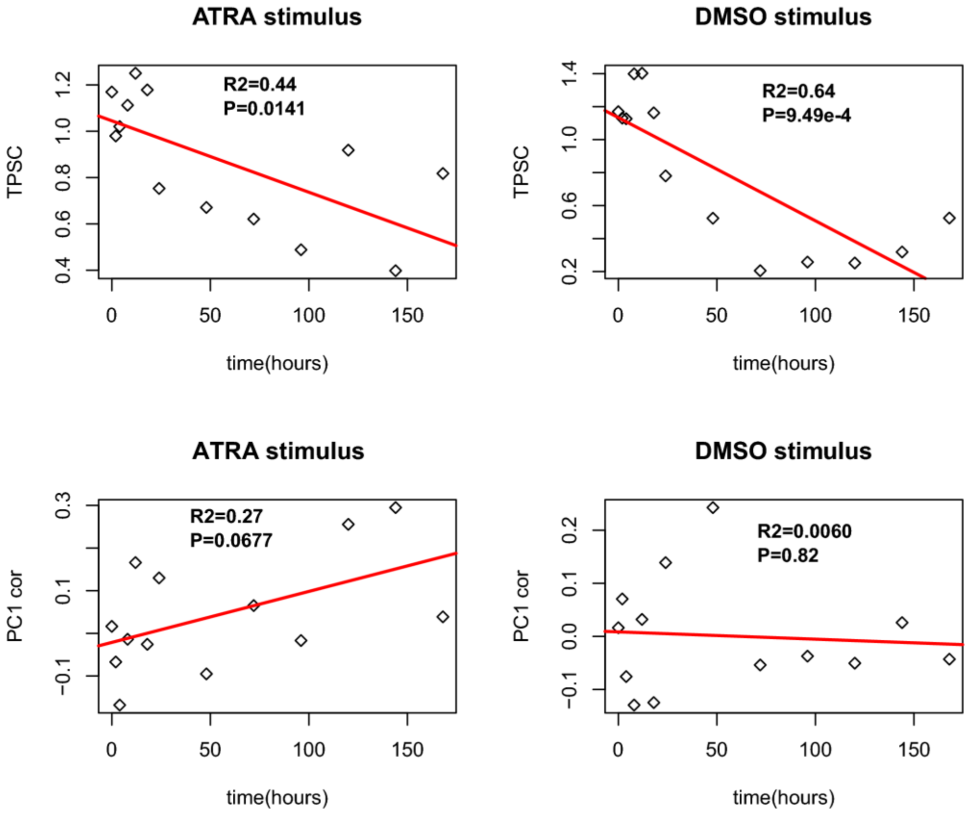

Supplementary Figure S22: As Supplementary Figure S21, but for the pluripotency signature of Palmer et al (30). The top panels show the corresponding t-test based pluripotency scores, while the lower panels show a pluripotency score estimated from a projection of each sample's expression profile onto the top principal component derived from Palmer's signature in this data set (see online Methods for detailed construction).

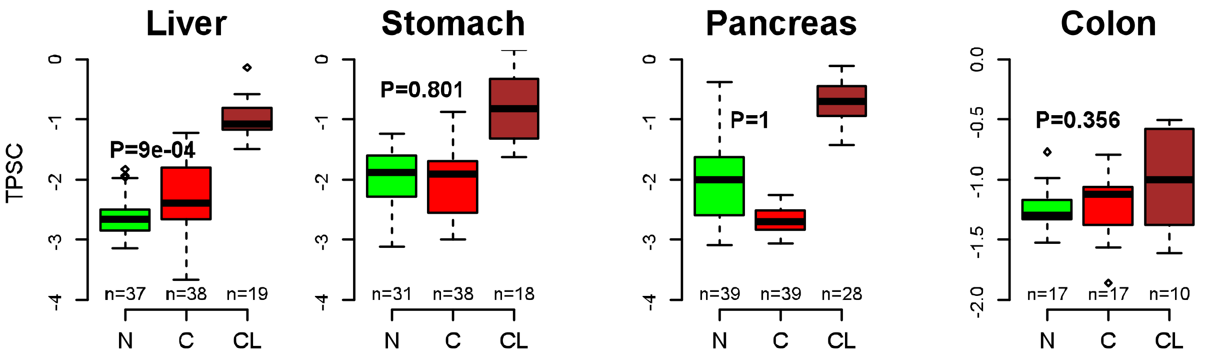

Supplementary Figure S23: Comparison of the t-test pluripotency score (TPSC) obtained from a pluripotency gene expression signature (29) in four tissue types (N=normal tissue, C=cancer tissue, CL=cancer cell line). All samples were done on the same Affy HG133 Plus2 arrays (online Methods). Number of samples in each group is given below boxes. In all cases, we provide the P-values from a one-tailed Wilcoxon rank sum test, testing the hypothesis that TPSC is higher in the undifferentiated cancer state compared to the differentiated normal tissue.

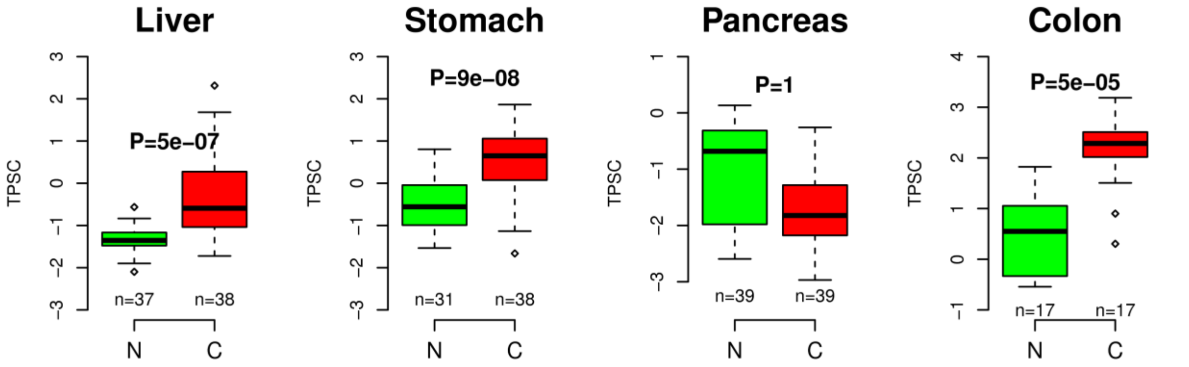

Supplementary Figure S24: Comparison of the TPSC pluripotency score obtained from Palmer's pluripotency signature (30) between normal (N) and cancer tissue (C) for different tissue types (20-23). All expression data was generated using Affymetrix HG-U133 Plus2 arrays. One tailed Wilcoxon rank sum test P-values are given, testing for higher scores in cancer. Observe how the TPSC score "breaks down" in the pancreatic set, predicting a lower TPSC score in the cancer tissue. Also note how the TPSC score, even though it is self-calibrating in each sample, varies significantly in value across tissue types, indicating that it is not a robust measure.

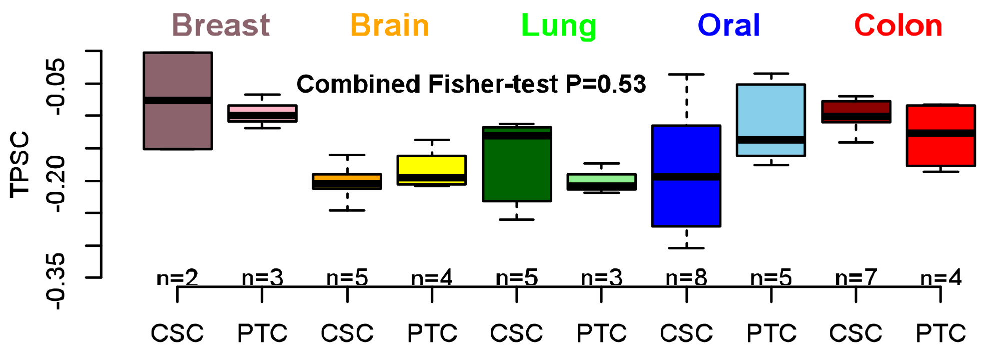

Supplementary Figure S25: Comparison of the pluripotency score derived from Mikkelsen's 19-gene signature between putative cancer stem cells (CSC) and their parental tumour cell lines (PTC) for five different tissue types. Expression data is from Yu et al (28) and was generated using Affymetrix HG-U133 Plus 2 arrays. Combined Fisher t-test P-value is given.

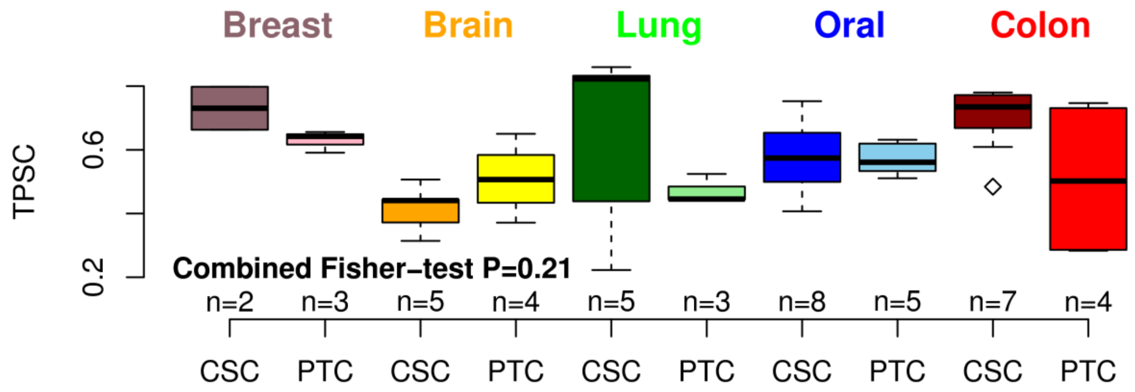

Supplementary Figure S26: Comparison of the TPSC pluripotency score obtained from Palmer's pluripotency signature (30) between putative cancer stem cells (CSC) and their parental tumour cell lines (PTC) across five different tissue types. Expression data is from Yu et al (28) and was generated using Affymetrix HG-U133 Plus 2 arrays. The p-value of a combined Fisher t-test is reported.

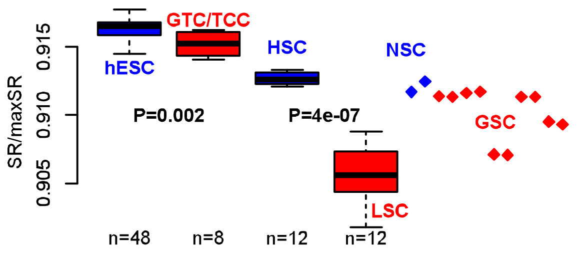

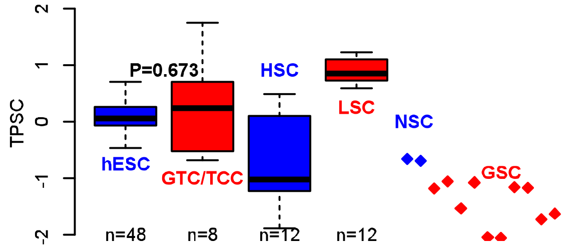

Supplementary Figure S27: Network entropy is lower in cancer stem cells than normal stem cells: Upper panel: Normalised network entropy rates of human embryonic stem cells (hESCs,n=48) and combined teratocarcinoma (n=5) / germ tumour (n=3) cell lines from the stem cell matrix compendium (all deemed pluripotent), as well as of 12 hematopoietic stem cells (HSC) and 12 CD34+ chronic myeloid leukemic stem cells (LSC) (Supplementary Materials). Wilcoxon rank sum test P-value between the pluripotent normal (hESC) and cancer (GTC/TCC) cells is given, as well as between the HSCs and LSCs. Finally, also included are the normalised network entropy rates (SR/maxSR) of a neural stem cell line (NSC) (in duplicate, blue) compared to that of four different MGG glioma stem cells (red, with replicates) (Supplementary Materials), as indicated. Lower panel: As upper panel but for the TPSC score derived from Mikkelsen's 19-gene pluripotency signature.

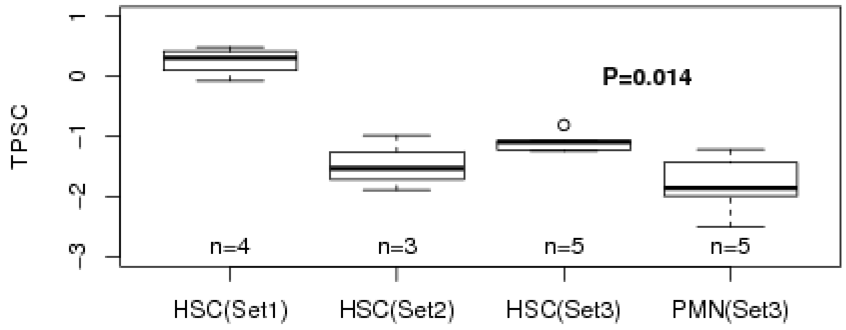

Supplementary Figure S28: The TPSC pluripotency score (derived from Mikkelsen's 19-gene signature) across 3 different data sets (see Supplementary Materials) profiling HSCs using the same Affymetrix U133A Plus 2 arrays. One of the data sets also contained polymorpho-neutrophils (PMNs). Wilcoxon-rank sum test P-value is between HSCs and PMNs of Set3. Observe how HSCs exhibit wild variations across the 3 data sets, with HSC(Set2) showing similar values to PMNs(Set3), in stark contrast to the entropy rate (compare with Supplementary Figure S11).

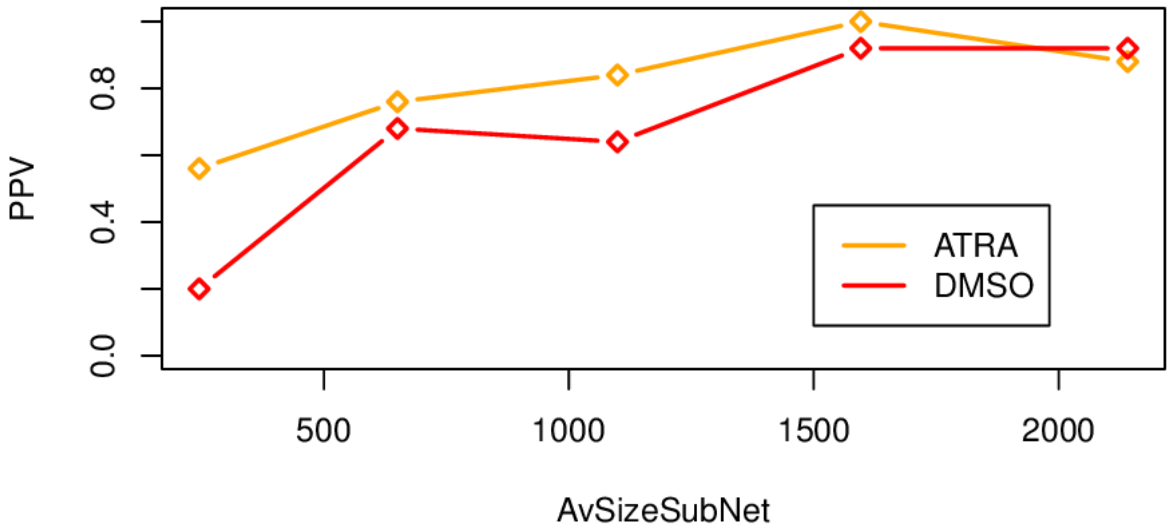

Supplementary Figure S29: Assessment of network entropy to exhibit significant linear decreases with differentiation stage in the HL60 to neutrophil time course differentiation data set (19), under random subsampling of the integrated expression PIN. Specifically, the x-axis labels the average size of maximally connected subnetworks obtained by randomly subsampling 500, 1000, 1500, 2000 and 2500 nodes from the full 5500 integrated PIN. The y-axis shows the corresponding average positive predictive value (PPV) that the resulting network entropy computed over the maximally connected component decreases significantly with time. In each case, averages were estimated over 25 subsampled subnetworks. As observed, when sampling 2000 nodes, resulting in max.connected subnetworks of size > 1500, we can see that in over 80% of subnetworks that we are still able to find a linear decrease of network entropy with time.

References:

1. Muller FJ*, et al.* (2008) Regulatory networks define phenotypic classes of human stem cell lines. *Nature* 455(7211):401-405.
2. Nazor KL*, et al.* (2012) Recurrent variations in DNA methylation in human pluripotent stem cells and their differentiated derivatives. *Cell stem cell* 10(5):620-634.
3. Barrett T*, et al.* (2013) NCBI GEO: archive for functional genomics data sets--update. *Nucleic acids research* 41(Database issue):D991-995.
4. Barberi T, Willis LM, Socci ND, & Studer L (2005) Derivation of multipotent mesenchymal precursors from human embryonic stem cells. *PLoS medicine* 2(6):e161.
5. Giraud-Triboult K*, et al.* (2011) Combined mRNA and microRNA profiling reveals that miR-148a and miR-20b control human mesenchymal stem cell phenotype via EPAS1. *Physiological genomics* 43(2):77-86.
6. Tanabe S*, et al.* (2008) Gene expression profiling of human mesenchymal stem cells for identification of novel markers in early- and late-stage cell culture. *Journal of biochemistry* 144(3):399-408.
7. Wagner W*, et al.* (2008) Replicative senescence of mesenchymal stem cells: a continuous and organized process. *PloS one* 3(5):e2213.

8. Larson BL, Ylostalo J, & Prockop DJ (2008) Human multipotent stromal cells undergo sharp transition from division to development in culture. *Stem Cells* 26(1):193-201.
9. Avery K, Avery S, Shepherd J, Heath PR, & Moore H (2008) Sphingosine-1-phosphate mediates transcriptional regulation of key targets associated with survival, proliferation, and pluripotency in human embryonic stem cells. *Stem cells and development* 17(6):1195-1205.
10. Ebert AD*, et al.* (2009) Induced pluripotent stem cells from a spinal muscular atrophy patient. *Nature* 457(7227):277-280.
11. Yu J*, et al.* (2009) Human induced pluripotent stem cells free of vector and transgene sequences. *Science* 324(5928):797-801.
12. Bock C*, et al.* (2011) Reference Maps of human ES and iPS cell variation enable high-throughput characterization of pluripotent cell lines. *Cell* 144(3):439-452.
13. Mrugala D*, et al.* (2009) Gene expression profile of multipotent mesenchymal stromal cells: Identification of pathways common to TGFbeta3/BMP2-induced chondrogenesis. *Cloning and stem cells* 11(1):61-76.
14. Hamidouche Z*, et al.* (2009) Priming integrin alpha5 promotes human mesenchymal stromal cell osteoblast differentiation and osteogenesis. *Proceedings of the National Academy of Sciences of the United States of America* 106(44):18587-18591.
15. Payton JE*, et al.* (2009) High throughput digital quantification of mRNA abundance in primary human acute myeloid leukemia samples. *The Journal of clinical investigation* 119(6):1714-1726.
16. Krejci O*, et al.* (2008) p53 signaling in response to increased DNA damage sensitizes AML1-ETO cells to stress-induced death. *Blood* 111(4):2190-2199.
17. Majeti R*, et al.* (2009) Dysregulated gene expression networks in human acute myelogenous leukemia stem cells. *Proceedings of the National Academy of Sciences of the United States of America* 106(9):3396-3401.
18. Watkins NA*, et al.* (2009) A HaemAtlas: characterizing gene expression in differentiated human blood cells. *Blood* 113(19):e1-9.
19. Huang S, Eichler G, Bar-Yam Y, & Ingber DE (2005) Cell fates as high-dimensional attractor states of a complex gene regulatory network. *Physical review letters* 94(12):128701.
20. Wurmbach E*, et al.* (2007) Genome-wide molecular profiles of HCV-induced dysplasia and hepatocellular carcinoma. *Hepatology* 45(4):938-947.
21. Badea L, Herlea V, Dima SO, Dumitrascu T, & Popescu I (2008) Combined gene expression analysis of whole-tissue and microdissected pancreatic ductal adenocarcinoma identifies genes specifically overexpressed in tumor epithelia. *Hepato-gastroenterology* 55(88):2016-2027.
22. Khamas A*, et al.* (2012) Screening for epigenetically masked genes in colorectal cancer Using 5-Aza-2'-deoxycytidine, microarray and gene expression profile. *Cancer genomics & proteomics* 9(2):67-75.
23. D'Errico M*, et al.* (2009) Genome-wide expression profile of sporadic gastric cancers with microsatellite instability. *Eur J Cancer* 45(3):461-469.
24. Barretina J*, et al.* (2012) The Cancer Cell Line Encyclopedia enables predictive modelling of anticancer drug sensitivity. *Nature* 483(7391):603-607.
25. Rheinbay E*, et al.* (2013) An aberrant transcription factor network essential for Wnt signaling and stem cell maintenance in glioblastoma. *Cell reports* 3(5):1567-1579.
26. Pollard SM*, et al.* (2009) Glioma stem cell lines expanded in adherent culture have tumor-specific phenotypes and are suitable for chemical and genetic screens. *Cell stem cell* 4(6):568-580.
27. Zhang B*, et al.* (2010) Effective targeting of quiescent chronic myelogenous leukemia stem cells by histone deacetylase inhibitors in combination with imatinib mesylate. *Cancer cell* 17(5):427-442.

28. Yu YH, *et al.* (2012) Network biology of tumor stem-like cells identified a regulatory role of CBX5 in lung cancer. *Scientific reports* 2:584.
29. Mikkelsen TS, *et al.* (2008) Dissecting direct reprogramming through integrative genomic analysis. *Nature* 454(7200):49-55.
30. Palmer NP, Schmid PR, Berger B, & Kohane IS (2012) A gene expression profile of stem cell pluripotentiality and differentiation is conserved across diverse solid and hematopoietic cancers. *Genome biology* 13(8):R71.
31. Cerami EG, *et al.* (2011) Pathway Commons, a web resource for biological pathway data. *Nucleic acids research* 39(Database issue):D685-690.
32. Prasad TS, Kandasamy K, & Pandey A (2009) Human Protein Reference Database and Human Proteinpedia as discovery tools for systems biology. *Methods Mol Biol* 577:67-79.
33. Kandasamy K, *et al.* (2010) NetPath: a public resource of curated signal transduction pathways. *Genome biology* 11(1):R3.
34. Komurov K & Ram PT (2010) Patterns of human gene expression variance show strong associations with signaling network hierarchy. *BMC systems biology* 4:154.
35. Dennis G, Jr., *et al.* (2003) DAVID: Database for Annotation, Visualization, and Integrated Discovery. *Genome biology* 4(5):P3.
36. Teschendorff AE & Severini S (2010) Increased entropy of signal transduction in the cancer metastasis phenotype. *BMC systems biology* 4:104.
37. Gomez-Gardenes J & Latora V (2008) Entropy rate of diffusion processes on complex networks. *Physical review. E, Statistical, nonlinear, and soft matter physics* 78(6 Pt 2):065102.
38. Manke T, Demetrius L, & Vingron M (2006) An entropic characterization of protein interaction networks and cellular robustness. *Journal of the Royal Society, Interface / the Royal Society* 3(11):843-850.
39. Ben-Porath I, *et al.* (2008) An embryonic stem cell-like gene expression signature in poorly differentiated aggressive human tumors. *Nature genetics* 40(5):499-507.
40. Mar JC & Quackenbush J (2009) Decomposition of gene expression state space trajectories. *PLoS computational biology* 5(12):e1000626.
